# Mesenteric artery calcium scoring: a potential screening method for chronic mesenteric ischemia

**DOI:** 10.1007/s00330-020-07530-0

**Published:** 2020-12-02

**Authors:** Luke G. Terlouw, Desirée van Noord, Theo van Walsum, Marco J. Bruno, Adriaan Moelker

**Affiliations:** 1 Department of Gastroenterology and Hepatology, Erasmus MC University Medical Center, Dr. Molewaterplein 40, Rotterdam, 3015 GD The Netherlands; 2 Department of Radiology and Nuclear Medicine, Erasmus MC University Medical Center, Rotterdam, The Netherlands; 3 Department of Gastroenterology and Hepatology, Franciscus Gasthuis & Vlietland, Rotterdam, The Netherlands; 4 Biomedical Imaging Group Rotterdam, Department of Radiology and Nuclear Medicine, Erasmus MC University Medical Center, Rotterdam, The Netherlands

**Keywords:** Atherosclerosis, Diagnostic imaging, Calcium score, Mesenteric arteries, Observer variation

## Abstract

**Objective:**

A practical screening tool for chronic mesenteric ischemia (CMI) could facilitate early recognition and reduce undertreatment and diagnostic delay. This study explored the ability to discriminate CMI from non-CMI patients with a mesenteric artery calcium score (MACS).

**Methods:**

This retrospective study included CTAs of consecutive patients with suspected CMI in a tertiary referral center between April 2016 and October 2019. A custom-built software module, using the Agatston definition, was developed and used to calculate the MACS for the celiac artery (CA), superior mesenteric artery (SMA), and inferior mesenteric artery. Scoring was performed by two blinded observers. Interobserver agreement was determined using 39 CTAs scored independently by both observers. CMI was defined as sustained symptom improvement after treatment. Non-CMI patients were patients not diagnosed with CMI after a diagnostic workup and patients not responding to treatment.

**Results:**

The MACS was obtained in 184 patients, 49 CMI and 135 non-CMI. Interobserver agreement was excellent (intraclass correlation coefficient 0.910). The MACS of all mesenteric arteries was significantly higher in CMI patients than in non-CMI patients. ROC analysis of the combined MACS of CA + SMA showed an acceptable AUC (0.767), high sensitivity (87.8%), and high NPV (92.1%), when using a ≥ 29.7 CA + SMA MACS cutoff. Comparison of two CTAs, obtained in the same patient at different points in time with different scan and reconstruction parameters, was performed in 29 patients and revealed significant differences in MACSs.

**Conclusion:**

MACS seems a promising screening method for CMI, but correction for scan and reconstruction parameters is warranted.

**Key Points:**

*• A mesenteric artery calcium score obtained in celiac artery and superior mesenteric artery has a high negative predictive value for chronic mesenteric ischemia and could serve as a screening tool.*

*• Interobserver agreement of the mesenteric artery calcium score is excellent.*

*• Scan and reconstruction parameters influence the mesenteric artery calcium score and warrant the development of a method to correct for these parameters.*

## Introduction

Postprandial abdominal pain and weight loss caused by fear of eating are incapacitating complaints associated with chronic mesenteric ischemia (CMI) [1, 2]. When left untreated, CMI has been reported to progress to acute mesenteric ischemia in 26–67% of patients [3, 4]. A recent study reported an overall annual CMI incidence of 9.2 per 100,000 inhabitants [5]. As previously shown, this study also reported atherosclerotic CMI (7.3 per 100,000) to be the greatest contributor to the overall incidence [5–7]. The incidence of atherosclerotic CMI showed a rising trend over the study period, suggesting that atherosclerotic CMI is an upcoming health issue. European experts agree that CMI still remains an underdiagnosed and undertreated disease, which is mainly caused by a lack of knowledge and awareness among physicians [8]. This observation is supported by a substantial diagnostic delay of approximately 6 months after onset of complaints [5, 9].

An easy to use non-invasive screening tool for CMI could facilitate early recognition and increase awareness for CMI, reducing undertreatment, diagnostic delay, and progression to acute mesenteric ischemia. The Agatston coronary artery calcium score (CACS) has been used to assess the risk of coronary artery disease and aid in clinical decision-making [10, 11]. A CACS of 0 has been shown to have a very high negative predictive value (NPV) of coronary artery disease [12]. A mesenteric artery calcium score (MACS) based on the same principles as the CACS could serve as a potential first test to identify patients in whom CMI is unlikely and identify patients in whom a further diagnostic workup is warranted. This study aimed to explore the ability to discriminate CMI patients from patients without CMI by using a MACS.

## Methods

### Study design

This single-center retrospective cohort study included consecutive patients analyzed for suspected CMI in a specialized tertiary referral center between April 2016 and October 2019. Patients were eligible for inclusion when a computed tomography (CT)—not older than 12 months before first presentation—was available. Patients were excluded when they had undergone a previous mesenteric artery revascularization, when an anatomical variation with a common origin of the celiac artery (CA) and superior mesenteric artery (SMA) was present, or when the duration of clinical follow-up after mesenteric artery revascularization was less than 3 months. The local medical research ethics committee decided that the Medical Research Involving Human Subjects Act does not apply to this study (MEC-2018-1414). The investigators complied with the Helsinki declaration on research ethics. The STROBE checklist for cohort studies was used to write this manuscript [13].

### Diagnostic workup of chronic mesenteric ischemia

A standardized diagnostic workup was performed in all patients and consisted of an assessment of symptoms, computed tomography angiography (CTA), and when indicated assessment of mucosal ischemia by visible light spectroscopy (VLS) [14]. Results of the workup were discussed by an experienced multidisciplinary expert team consisting of gastroenterologists, interventional radiologists, and vascular surgeons. Patients were treated when a consensus diagnosis of CMI was established, which was based on presenting symptoms, imaging, VLS, and absence of a possible alternative diagnosis [15]. A definitive diagnosis of CMI was established when symptoms improved or resolved at three months after revascularization, or in case of chronic non-occlusive mesenteric ischemia during treatment with a vasodilator. Patients with a consensus diagnosis of no CMI or those initially labeled as CMI, but without improvement of symptoms after treatment, were classified as non-CMI. A definitive diagnosis of CMI was used for the primary outcome, which was to determine the ability of the MACS to discriminate patients with a definitive diagnosis of CMI from non-CMI patients.

### Calcium scoring tool

**Fig. 1:**
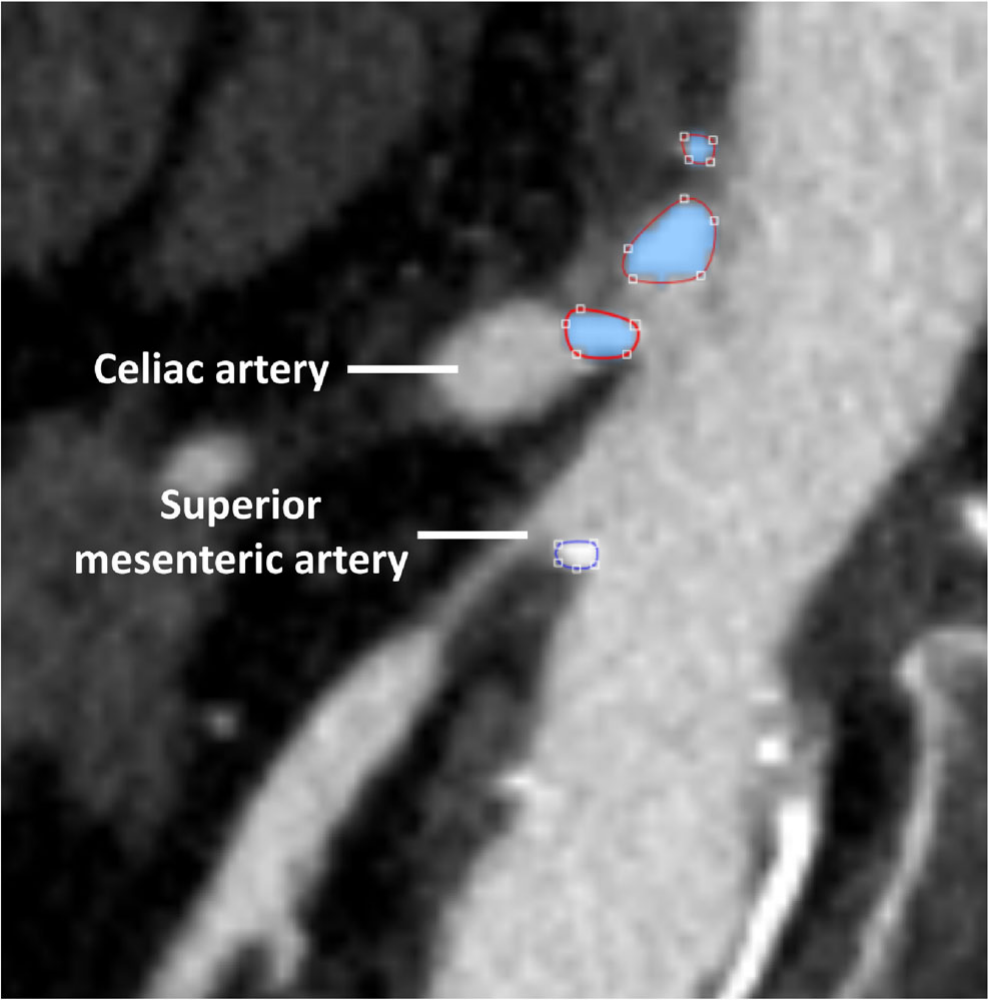
Regions of interest for calculation of the mesenteric artery calcium score on computed tomography angiography, using MeVisLab

The majority of the available CTs were contrast enhanced, which is explained by the fact that a CTA is indicated in the workup of CMI in order to assess vessel patency. When multiple series with different contrast enhancement phases were available, the series with the lowest slice thickness, generally the arterial contrast enhancement series, was used to calculate the MACS. Available semi-automated calcium scoring software can not differ calcified plaques from contrast; i.e., contrast is labeled as calcium, and therefore not suitable for use within this study. A custom-built software module in MeVisLab version 2.7.1 (MeVis Medical Solutions AG) was developed to identify calcified lesions by assigning hand drawn regions of interest on CTA (Fig. 1). The MACS was calculated according to the Agatston definition, which is calculated by multiplying the volume of a lesion with a density factor [10]. To assess the technical accuracy of the software, the CACS of six cardiac CTs without contrast enhancement analyzed by both Syngo.via (Siemens Healthcare) and by MeVisLab were compared.

### Mesenteric artery calcium score

**Fig. 2:**
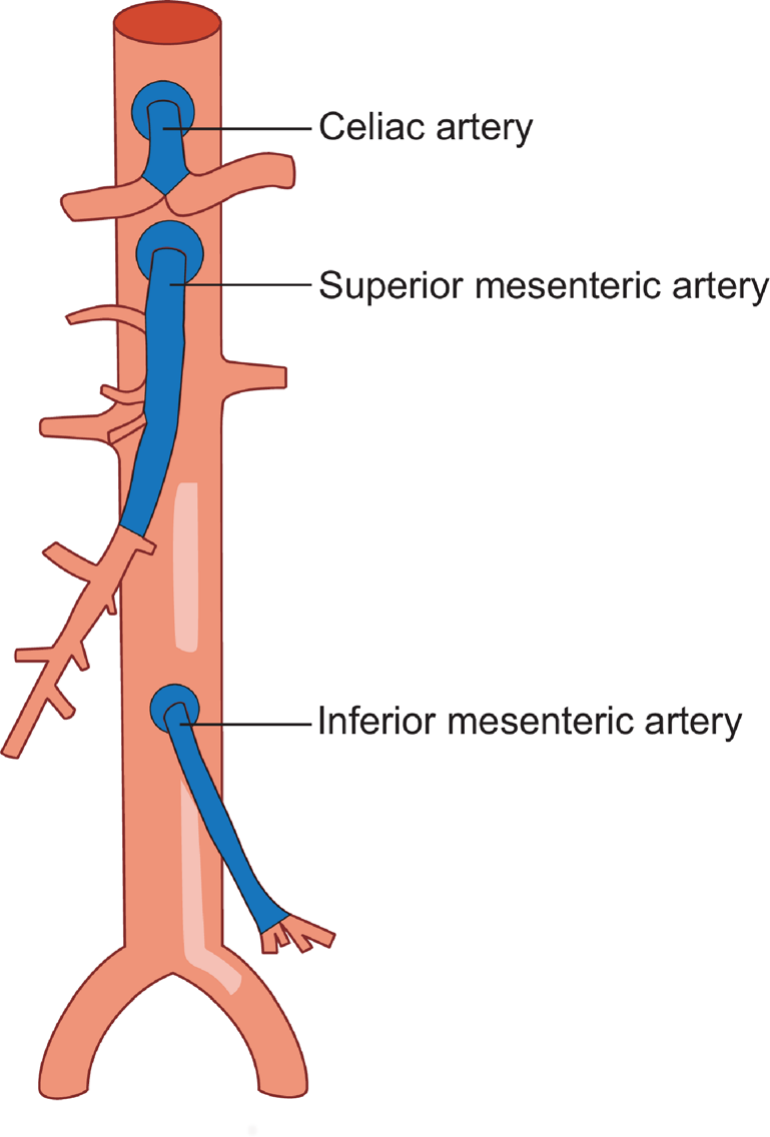
Regions included in the mesenteric artery calcium score

The MACS was obtained for all three mesenteric arteries individually; the total MACS was the sum of the MACS of the CA, SMA, and inferior mesenteric artery (IMA). Mesenteric artery stenoses most frequently occur at the origin of a mesenteric artery and involve the aorta wall directly surrounding the origin; distal stenoses are less common [16, 17]. Assumptions were made to standardize the areas of calcium scoring. The estimated volume of a calcified lesion causing a stenosis at the vessels’ origin consisted of the volume of the lesion that was located within a circle with a radius of 1 time the diameter of the vessels’ origin (Fig. 2). Calcium scoring was performed from origin until bifurcation for CA, from origin until the first large jejunal artery for SMA, and from origin until left colic artery for IMA.

Calcium scoring was performed by two observers, an interventional radiologist with 9 years of experience and a trainee with little experience. Both observers were blinded to patient history and clinical outcome. The first 9 CTAs served as a training set and were reviewed by both observers; scoring was performed based on consensus. The following 39 CTAs were used to determine interobserver agreement and scored independently by both observers, while remaining blinded to each other’s results. A sample size of 13 CTAs would suffice for assessment of the intraclass correlation coefficient (ICC), when comparing results of two observers, while using an alpha of 0.05 and a beta of 0.1 [18]. However, an atherosclerotic stenosis is observed in approximately 43–53% of patients [5, 7]. The required sample size of 13 CTAs was tripled to 39 CTAs in order to include sufficient CTAs with calcified lesions, since interobserver variability is expected to be greater when scoring CTAs with calcifications. Interobserver agreement was assessed directly upon completion of the 39 CTAs. When the interobserver agreement was good (ICC 0.75 to 0.9) or excellent (ICC ≥ 0.9), the remaining CTAs would be scored by either one of the observers [19]. When the interobserver agreement was poor (ICC < 0.5) or moderate (ICC 0.5 to 0.75), the remaining CTAs would be scored by both observers.

### Variability scan protocols

The vast majority of patients were referred by other hospitals and CTA imaging had often been performed in the referring hospital, causing a large variety in the used scan protocols. A CTA, performed using a standardized mesenteric artery protocol, was repeated during the workup when the image quality was deemed insufficient to reliably assess mesenteric artery patency. When two scans of the same patient were available calcium scoring was performed on both scans. Both scans had to be performed within 12 months before presentation, since clinically significant progression of atherosclerosis was expected to be unlikely within this time window. The scan performed in the tertiary referral center was included in the analysis of the primary endpoint. A separate analysis was performed to determine whether imaging and reconstruction parameters influenced the MACS by comparing the MACS and CTA characteristics—i.e., slice thickness, pixel spacing, tube voltage, and contrast enhancement—of the two scans. The density of contrast enhancement was calculated by the sum of the mean and two times the standard deviation (sd) of the Hounsfield unit (HU) value measured within the center of the aorta at the level of the origin of the mesenteric artery.

### Data collection and statistical analysis

Data regarding medical history, presenting symptoms, presence of a mesenteric artery stenosis (defined as ≥ 50% luminal reduction), diagnosis, follow-up, CTA characteristics, density of contrast enhancement, and MACS were collected and pseudo anonymized with a unique code.

Statistical analyses were performed with R version 3.5.3 (R Foundation for Statistical Computing) using packages pROC and irr. Continuous variables were not equally distributed and, therefore, shown as median and interquartile range (IQR). Baseline characteristics of CMI and non-CMI patients were compared using chi-square or Fisher’s exact testing for categorical variables and Wilcoxon rank testing for continuous variables. The ICC was used to assess MACS interobserver agreement and correlation between the CACS calculated by Syngo.via and MeVisLab. Receiver operator characteristics curves were computed to define MACS cutoff values to discriminate CMI patients from non-CMI patients. The area under the curve (AUC) was used to quantify the discriminative ability of the MACS. An AUC of 0.5 suggests no discrimination; AUC of 0.7 to 0.8 acceptable discrimination; AUC of 0.8 to 0.9 excellent discrimination; and AUC ≥ 0.9 outstanding discrimination [20]. The sign test was used to compare the characteristics and MACS of two CTAs of the same patient (obtained at two different points in time).

## Results

During the study period, 203 patients were analyzed for suspected CMI. A total of 184 patients was included in the study. Reasons for exclusion were a previous mesenteric artery revascularization in 9 patients, a common origin of CA and SMA in 2 patients, and a follow-up duration of < 3 months after revascularization in 8 patients. A definitive diagnosis of CMI was established in 49 of the included patients, of whom 46 were diagnosed with atherosclerotic CMI, 2 with median arcuate ligament syndrome (MALS), and 1 with chronic non-occlusive mesenteric ischemia. The remaining 135 patients were classified as non-CMI.

### Baseline and CTA characteristics

CMI patients were significantly older (CMI 70 (65–75) vs. non-CMI 64 (56–70)) and significantly more often female (CMI 81.6% vs. non-CMI 65.9%). Comparison of risk factors of cardiovascular disease (CVD) showed that a history of CVD, a history of peripheral artery disease, and smoking were significantly more prevalent among CMI patients (Table 1). The presenting symptoms of weight loss, postprandial abdominal pain, diarrhea, and an adapted eating pattern were also significantly more prevalent in CMI patients.

**Table 1:** Baseline characteristics

Baseline characteristic	All patients	CMI patients	Non-CMI patients	*p* value
	(*N* = 184)	(*N* = 49)	(*N* = 135)	
Female gender	70.1%	81.6%	65.9%	*p* = 0.061
Age	67 (57–73)	70 (65–75)	64 (56–70)	*p* = 0.002
Follow-up (months)	6 (2–11)	10 (8–24)	3 (1–9)	*p* < 0.001
Risk factors				
Cardiovascular disease	44.0%	61.2%	37.8%	*p* = 0.008
Peripheral artery disease	20.7%	42.9%	12.6%	*p* < 0.001
Coronary artery disease	21.7%	26.5%	20.0%	*p* = 0.455
Cerebrovascular disease	12.5%	16.3%	11.1%	*p* = 0.488
Dyslipidemia	13.6%	16.3%	12.6%	*p* = 0.682
Hypertension	33.2%	34.7%	32.6%	*p* = 0.928
Diabetes	14.1%	20.4%	11.9%	*p* = 0.217
Family history of CVD	38.9%	44.4%	36.8%	*p* = 0.472
Smoking	66.7%	83.7%	60.3%	*p* = 0.005
Pack years	25 (11–44)	33 (20–49)	21 (4–40)	*p* = 0.028
Presenting symptoms				
Weight loss	64.2%	93.8%	53.4%	*p* < 0.001
Weight (kg)	64 (56–79)	61 (52–70)	66 (57–81)	*p* = 0.147
Body mass index	23 (20–27)	21 (19–26)	23 (20–27)	*p* = 0.114
Abdominal pain	91.3%	95.9%	89.6%	*p* = 0.243
Duration abdominal pain (months)	10 (4–24)	5 (3–12)	12 (6–24)	*p* = 0.047
Postprandial abdominal pain	63.5%	83.0%	55.8%	*p* = 0.002
Exercise-induced abdominal pain	37.8%	36.4%	38.3%	*p* = 1.000
Adapted eating pattern	55.5%	83.3%	46.4%	*p* < 0.001
Nausea	57.3%	63.4%	54.4%	*p* = 0.440
Diarrhea	21.2%	39.6%	14.5%	*p* = 0.001

Numerical variables are shown as median (interquartile range); *p* < 0.05 is considered statistically significant

*CMI*, chronic mesenteric ischemia; *CVD*, cardiovascular disease; *kg*, kilogram

CTA revealed no mesenteric artery stenosis in 56 (30.4%) patients, CA stenosis in 45 (24.5%) patients, SMA stenosis in 17 (9.2%) patients, IMA stenosis in 10 (5.4%) patients, CA and SMA stenosis in 18 (9.8%) patients, CA and IMA stenosis in 10 (5.4%) patients, SMA and IMA stenosis in 10 (5.4%) patients, and CA, SMA, and IMA stenosis in 18 (9.8%) patients (Table 2). The nature of the stenosis was atherosclerosis in 77.1% of patients, compression in 19.1%, iatrogenic in 2.3%, and a combination of atherosclerosis and compression in 1.5% of patients (Table 2). Comparison of the CTA characteristics of CMI and non-CMI patients did not show a significant difference in tube voltage, slice thickness, pixel spacing, or contrast density at the level of CA, SMA, or IMA.

**Table 2:** Location and nature of mesenteric artery stenosis

Stenosis characteristics	CMI patients	Non-CMI patients
	(*N* = 49)	(*N* = 135)
No significant stenosis	1 (1.8%)	55 (98.2%)
CA	6 (13.3%)	39 (86.7%)
SMA	3 (17.6%)	14 (82.4%)
IMA	0 (0.0%)	10 (100.0%)
CA and SMA	13 (72.2%)	5 (27.8%)
CA and IMA	2 (20.0%)	8 (80.0%)
SMA and IMA	8 (80.0%)	2 (20.0%)
CA, SMA, and IMA	16 (88.9%)	2 (11.1%)
Nature of stenosis	(*N* = 48)	(*N* = 83)
Atherosclerosis	46 (45.5%)	55 (54.5%)
Compression	2 (8.0%)	23 (92.0%)
Iatrogenic	0 (0.0%)	3 (100.0%)
Atherosclerosis and compression	0 (0.0%)	2 (100.0%)

The shown data are numbers and (proportions)

*CA*, celiac artery; *CMI*, chronic mesenteric ischemia; *IMA*, inferior mesenteric artery; *SMA*, superior mesenteric artery

### Calcium scoring software accuracy and interobserver agreement

The CACS on the cardiac CTs, calculated by Syngo.via and MeVisLab, showed an excellent correlation with an ICC of 0.998 (95% confidence interval (CI) 0.991–1.000). Proving technical accuracy after which the assessment of the MACS was commenced. MACS of the 39 CTAs scored by both observers showed an excellent correlation as well (ICC 0.910 (95% CI 0.834–0.951)). Hence, the remaining CTAs were scored by either one of the observers.

### Mesenteric artery calcium score

The MACSs of CA, SMA, IMA, CA + SMA, and the total MACS were all significantly higher in CMI patients than in non-CMI patients (Table 3). The ability to discriminate CMI patients from non-CMI patients using the MACS was assessed by calculating the AUC. MACSs of CA + SMA and total MACS showed the highest discriminative ability with acceptable AUCs of 0.767 (95% CI 0.686–0.847) and 0.770 (95% CI 0.690–0.851) respectively (Table 4). The best test characteristics for the identification of CMI patients were found when using a MACS cutoff of ≥ 29.7 for CA + SMA: sensitivity 87.8%, specificity 51.9%, positive predictive value (PPV) 39.8%, and negative predictive value (NPV) 92.1%. Seventy of the 184 (38%) patients had a MACS < 29.7 and no definitive diagnosis of CMI.

**Table 3:** Mesenteric artery calcium scores

MACS	CMI patients	Non-CMI patients	*p* value
	(*N* = 50)	(*N* = 134)	
CA	166 (71–510)	0 (0–136)	*p* < 0.001
SMA	463 (8–1130)	0 (0–130)	*p* < 0.001
IMA	21 (0–66)	0 (0–19)	*p* = 0.005
CA + SMA	832 (96–1803)	17 (0–278)	*p* < 0.001
Total	966 (149–1701)	31 (0–351)	*p* < 0.001

Calcium scores are shown as median (interquartile range); *p* < 0.05 is considered statistically significant

*CA*, celiac artery; *CMI*, chronic mesenteric ischemia; *IMA*, inferior mesenteric artery; *MACS*, mesenteric artery calcium score; *SMA*, superior mesenteric artery

**Table 4:** Test characteristics of the mesenteric artery calcium scores

MACS	AUC (95% CI)	Cutoff	Sensitivity (%)	Specificity (%)	PPV (%)	NPV (%)
CA	0.752 (0.674–0.830)	16.0	85.7	53.3	40.0	91.1
SMA	0.752 (0.668–0.835)	29.0	73.5	62.2	41.4	86.6
IMA	0.661 (0.570–0.753)	2.6	71.7	53.8	35.1	84.5
CA + SMA	0.767 (0.686–0.847)	29.7	87.8	51.9	39.8	92.1
Total	0.770 (0.690–0.851)	34.6	87.8	51.1	39.4	92.0

*AUC*, area under the curve; *CA*, celiac artery; *CI*, confidence interval; *CMI*, chronic mesenteric ischemia; *IMA*, inferior mesenteric artery; *MACS*, mesenteric artery calcium score; *NPV*, negative predictive value; *SMA*, superior mesenteric artery; *PPV*, positive predictive value

### Influence scan protocol on calcium score

A scan performed in both the tertiary referral center and the referring hospital was available for 29 patients. Comparison of the CTAs showed a significant difference in the used tube voltage and the contrast densities of the CTAs (Table 5). The slice thickness of the tertiary referral center CTAs was significantly thinner than the slice thickness of the CTAs of the referring hospital (tertiary referral center 0.8 (0.8–0.9) vs. referring hospital 3.0 (2.0–3.0)). When comparing the MACS of both CTAs, the MACS of SMA, CA + SMA, and the total MACS were all significantly higher when calculated on the CTA of the referring hospital.

**Table 5:** Comparison of CTA characteristics and calcium scores of CTAs of the same patient

CTA characteristics	Tertiary referral center	Referring hospital	*p* value
	(*N* = 29)	(*N* = 29)	
Tube voltage (kVp)			*p* = 0.001
70	5 (17.2%)	1 (3.4%)	
80	15 (51.7%)	4 (13.8%)	
90	2 (6.9%)	1 (3.4%)	
100	3 (10.3%)	11 (37.9%)	
120	4 (13.8%)	12 (41.4%)	
Slice thickness (mm)	0.8 (0.8–0.9)	3.0 (2.0–3.0)	*p* < 0.001
Pixel spacing	0.7 (0.6–0.7)	0.7 (0.6–0.7)	*p* = 0.845
Contrast density			
Aorta level CA	625.1 (477.6–717.5)	213.3 (186.7–400.4)	*p* < 0.001
Aorta level SMA	639.9 (518.5–737.5)	207.4 (180.7–402.4)	*p* < 0.001
Aorta level IMA	637.3 (500.0–769.3)	267.2 (193.1–489.3)	*p* < 0.001
MACS			
CA	114 (0–320)	192 (0–435)	*p* = 0.078
SMA	330 (0–577)	352 (0–945)	*p* = 0.012
IMA	9 (0–51)	22 (0–59)	*p* = 0.824
CA + SMA	519 (4–966)	728 (0–1375)	*p* = 0.017
Total	566 (7–966)	798 (24–1427)	*p* = 0.023

Numerical variables are shown as median (interquartile range); *p* < 0.05 is considered statistically significant

*CA*, celiac artery; *CMI*, chronic mesenteric ischemia; *CTA*, computed tomography angiography; *IMA*, inferior mesenteric artery; *kVp*, kilovoltage peak; *MACS*, mesenteric artery calcium score; *mm*, millimeter; *SMA*, superior mesenteric artery

## Discussion

This study is the first to report the use of a MACS to discriminate CMI patients from non-CMI patients. A combined MACS of CA + SMA showed most promising for this purpose with an AUC of 0.767 and a high NPV (92.1%), using a ≥ 29.7 cutoff for CA + SMA. Interobserver agreement of the MACS was excellent (ICC 0.910). The used scan protocol and reconstruction parameters (slice thickness, tube voltage, and contrast enhancement) influenced the calculated MACS.

Limited awareness for CMI and lack of knowledge among physicians cause diagnostic delays. An easy to use screening tool for CMI could shorten this diagnostic delay, but is unavailable. The CA + SMA calcium score seems able to discriminate CMI patients from non-CMI patients. The discriminative ability was acceptable (AUC 0.767) and the sensitivity (87.8%) and NPV (92.1%) were high. Even though the MACS was calculated on CTA and reconstruction parameters varied between scans, the MACS still approximates the test characteristics of a CACS of 0 (sensitivity 99–100%, NPV 97–100%), indicating that the CA + SMA MACS is a promising screening method [12]. A second reason to opt for use of the CA + SMA MACS is the good recognizability of the origin of the CA and SMA. While the identification of the origin of the IMA can be more cumbersome, especially on non-contrast-enhanced CT, and the addition of the IMA to the MACS seems of limited value. Calculation of the CA + SMA MACS on CTs of patients with weight loss or postprandial abdominal pain in whom the diagnosis CMI is considered and, in whom an alternative diagnosis is not found, is an attractive clinical application of this score. A low calcium score effectively rules out CMI, implying that a CTA would not be indicated in approximately 38% of patients, which is advantageous to patients with impaired renal function. CMI is more probable in case of an increased CA + SMA MACS, warranting a diagnostic workup, including a CTA with ≤ 1-mm slice thickness to assess vessel patency [8].

The currently used software enabled calcium scoring on contrast-enhanced CT. The interobserver agreement was excellent (ICC 0.910), even though regions of interest were drawn by hand and were performed by an experienced interventional radiologist and a trainee, suggesting that the MACS on CTA does not depend on the scoring radiologist. The 39 CTs scored by both observers included too few non-contrast-enhanced CTs to reliably assess the effect of contrast enhancement on the interobserver agreement of the MACS. Observers could have falsely included contrast at the borders of the lesion and could have missed calcifications with a low intensity that were concealed by high intensity of contrast enhancement, which influences reliability of the MACS. The current necessity to draw regions of interest by hand is time consuming, especially when the slice thickness is ≤ 1 mm. Development of (semi-)automated calcium scoring software, such as currently used for the CACS, should be validated for several levels of intensity of contrast enhancement in order to solve the limitation of contrast enhancement and make mesenteric artery calcium scoring fast and feasible for clinical practice.

This retrospective study is a first attempt to identify CMI patients using a MACS and should be considered a proof of principle. The high NPV indicates that the MACS could potentially be a much desired screening tool for CMI. However, suggesting a suitable cutoff for clinical use is premature due to limitations of the current study, such as the large variation in CTA characteristics of the included scans, which influenced the MACS when comparing two CTAs performed in one patient. The influence of tube voltage, slice thickness, and reconstruction kernel on the calcium score has already been described in CACS literature [21–23]. The influence of tube voltage on the CACS has recently been studied by Booij et al; they showed that CACSs on a phantom significantly differed when tube voltage increased [21]. Qian et al compared the volume of coronary calcium on 0.5-mm and 3.0-mm slice thickness reconstructions, using intravascular ultrasound with radiofrequency backscatter-virtual histology as a reference [22]. Reconstructions of 0.5-mm reduced the overestimation of calcium volume as compared to conventional 3.0-mm reconstructions and had a higher sensitivity (0.5-mm 94%, 3.0 mm-56%), but lower specificity (0.5-mm 50%, 3.0-mm 93%). Mantini et al reported that the CACS on 0.5-mm slice thickness reconstructions resulted in significant reclassification of the cardiovascular risk when compared with standard 3.0-mm reconstructions [23]. The use of reconstructions with a different reconstruction kernel also resulted in significant reclassification of the cardiovascular risk, showing that variation in the used reconstruction parameters can influence clinical decision-making. The results of these studies support the observed influence of scan and reconstruction parameters on the MACS, underlining that correction factors for slice thickness, tube voltage, and contrast enhancement are needed to set a reliable MACS cutoff that is feasible for use in clinical practice. Correction factors could be calculated by a phantom study using different slice thicknesses, tube voltages, and intensities of contrast enhancement.

Other possible limitations of this study are related to the included patient population and retrospective study design. First, all included patients were referred because of suspected CMI based on typical symptoms and/or mesenteric artery stenosis, resulting in a population with a higher pretest probability of CMI. However, the prevalence of CVD risk factors and typical symptoms still differed significantly between CMI and non-CMI patients, suggesting that the predictive value of MACS could be improved by relating the MACS to history and clinical symptoms. Hence, the addition of the MACS to an existing CMI score chart could improve the accuracy of the predicted CMI probability and possibly prevent a missed diagnosis of CMI in patients with a non-calcified mesenteric artery stenosis [7]. Second, our inclusion strategy resulted in the inclusion of two MALS patients. These patients had a MACS of 0, since MALS is caused by CA compression and not by atherosclerosis. MALS is typically seen in patients of younger age, without cardiovascular risk factors [2, 24]. Non-atherosclerotic causes of CMI, such as MALS or vasculitis, are more probable in patients of younger age. Selection of patients based on age, e.g., ≥ 40 years of age, could be a viable strategy to improve the sensitivity of the MACS and avoid false-negative tests. Third, the follow-up duration of non-CMI patients was shorter than the follow-up duration of CMI patients. Non-CMI patients could have been treated in another center after their last outpatient clinic visit, although this is unlikely, since there is only one other mesenteric ischemia expert center in the country. Fourth, the number of included patients was too low to allow subgroup analysis, e.g., to determine MACS cutoffs for each level of tube voltage.

This proof of principle study demonstrates the ability to discriminate CMI patients from non-CMI patients using the MACS (AUC 0.767). The MACS could be a promising screening tool for CMI with a high sensitivity (87.8%) and high NPV (92.0%), despite the mentioned limitations. These results suggest that a low MACS rules out CMI and an increased MACS warrants and justifies further diagnostic workup with a ≤ 1-mm CTA [8]. Correction for scan and reconstruction parameters is needed, since we observed significant differences in MACSs scored on two CTAs of the same patient acquired with a different scan and/or reconstruction protocol. Implementation and validation of an algorithm to correct for scan and reconstruction parameters seem to be the next steps to further evaluate the clinical promise and applicability of this tool.

## Abbreviations

AUC: Area under the curve
CA: Celiac artery
CACS: Coronary artery calcium score
CI: Confidence interval
CMI: Chronic mesenteric ischemia
CT: Computed tomography
CTA: Computed tomography angiography
CVD: Cardiovascular disease
HU: Hounsfield unit
ICC: Intraclass correlation coefficient
IMA: Inferior mesenteric artery
IQR: Interquartile range
kVp: Kilovoltage peak
MACS: Mesenteric artery calcium score
MALS: Median arcuate ligament syndrome
NPV: Negative predictive value
PPV: Positive predictive value
Sd: Standard deviation
SMA: Superior mesenteric artery
VLS: Visible light spectroscopy
